# Association between ALT/AST and Muscle Mass in Patients with Type 2 Diabetes Mellitus

**DOI:** 10.1155/2022/9480228

**Published:** 2022-10-14

**Authors:** Wenjie Ma, Wenchao Hu, Yuantao Liu, Lanjie He

**Affiliations:** Department of Endocrinology, Qilu Hospital (Qingdao), Cheeloo College of Medicine, Shandong University, Qingdao, Shandong, China

## Abstract

**Objective:**

The alanine aminotransferase/aspartate aminotransferase (ALT/AST) ratio is thought to be related to metabolic disorders and insulin resistance. Type 2 diabetes mellitus (T2DM) is a high-risk population for low muscle mass. This study was performed to evaluate the association between ALT/AST and muscle mass in subjects with T2DM.

**Method:**

This cross-sectional study enrolled 1068 subjects (566 males and 502 females) with T2DM. General information, medical history, and blood samples were collected. Skeletal muscle index (SMI) was detected using dual-energy X-ray absorptiometry. Logistic regression analysis was utilized to determine the correlation of ALT/AST and low muscle mass in subjects with T2DM. Multiple linear regression analysis was utilized to evaluate the association between ALT/AST, SMI and other metabolic characteristics.

**Result:**

Of all subjects, 115 men (20.3%) and 71 women (14.1%) presented low muscle mass. ALT/AST was related to an increased risk for low muscle mass in both genders. Multiple linear regression analysis displayed that SMI was negatively associated with ALT/AST, age, glycosylated hemoglobin (HbA1c), and high-density lipoprotein cholesterol (HDL) in male group. While in female group, SMI was positively associated with systolic blood pressure (SBP) and negatively associated with ALT/AST and age. Furthermore, ALT/AST was associated with age and BMI in both genders.

**Conclusion:**

ALT/AST was negatively associated with muscle mass in subjects with T2DM.

## 1. Introduction

Sarcopenia is a syndrome featured with the gradual and widespread decline in skeletal muscle mass and function [1]. Skeletal muscle plays a vital role in posture and movement. It is also essential for glucose uptake and energy metabolism. Skeletal muscle mass gradually decreases with aging, resulting in impaired muscle strength and mobility [2]. Sarcopenia has been demonstrated to be related to movement disorders, falls, fractures, disabilities, and metabolic disorders [3]. Type 2 diabetes mellitus (T2DM) is a very common metabolic disease, which is widely distributed all over the world. In 2021, the number of adults with diabetes worldwide reached 537 million. Earlier studies have shown that T2DM is associated with an increased risk for sarcopenia [4–6]. The risk of sarcopenia in subjects with T2DM was obviously elevated compared to subjects with normal blood glucose. According to a Korean study, the risk of sarcopenia in diabetic patients was two to four times compared with that in nondiabetic patients [7]. The incidence of sarcopenia was 28.4% in T2DM group and 18.7% in control group reported by Veronese et al. [8]. In view of the adverse impact of low muscle mass or sarcopenia on patients, timely identification and intervention is especially important. One potential approach is to look for probable risk factors or biomarkers for sarcopenia.

Serum alanine aminotransferase/aspartate aminotransferase (ALT/AST) ratio has been commonly used as an indicator for liver damage. Over the past few years, several researches showed that ALT/AST was associated with insulin resistance and metabolic disorders [9–12]. Zou et al. showed that ALT/AST was associated with new-onset nonalcoholic fatty liver disease [13]. Song et al. reported that ALT/AST levels in early pregnancy were correlated with the incidence of gestational diabetes [14]. A large study in western China showed that elevated serum AST/ALT was related to an increased prevalence of sarcopenia among people over 50 years of age [15].

So far, the studies about the association between ALT/AST and muscle mass in people with T2DM are limited. The present study was performed to analyze the correlation between ALT/AST and muscle mass in T2DM patients.

## 2. Materials and Methods

### 2.1. Subjects

This cross-sectional study was performed in the Endocrinology department of Qilu Hospital (Qingdao) from September 2017 to September 2019. A total of 1068 patients with T2DM were included in our study. All selected participants are ≥20 years old. Exclusion criteria include patients with pregnancy, severe liver, kidney or cardiovascular diseases, malignant tumor, and disability. This research was approved by the hospital ethics committee and carried out according to the Declaration of Helsinki.

### 2.2. The Criterion of Low Muscle Mass

Skeletal muscle index (SMI) was detected by the dual-energy X-ray absorptiometry (Hologic Discovery A). All subjects were operated by the same technician. SMI is equal to the skeletal muscle mass of limbs in kilograms divided by the square of height in meters (kg/m^2^). According to the consensus of Asian Working Group for Sarcopenia, the standard of low muscle mass is SMI less than 7.0 kg/m^2^ for men or 5.4 kg/m^2^ for women [16].

### 2.3. Measurements

The anthropometry, clinical, and laboratory analysis were carried out. Height, weight, systolic blood pressure (SBP), and diastolic blood pressure (DBP) were measured in all subjects. Body mass index (BMI) was computed according to the formula of weight in kilograms divided by the square of height in meters (kg/m^2^). After fasting overnight, blood specimens were collected from all participants. ALT, AST, fasting blood glucose (FPG), glycosylated hemoglobin (HbA1c), triglyceride (TG), high-density lipoprotein cholesterol (HDL), low-density lipoprotein cholesterol (LDL), and total cholesterol (TC) were detected in all subjects.

### 2.4. Statistical Analysis

Make use of Statistical Products and Service Solutions (SPSS for Windows, 22nd edition, Chicago, Illinois) for analysis. Continuous variables were tested for normality of distribution. Variables with approximately normal distributions were described by mean ± standard deviation (SD) and those with skewed distributions were described by median and 25 to 75th percentile. Categorical variables were described by number and proportion. The unpaired *t*-test or Mann–Whitney test was used for continuous variables and chi-square test was used for categorical variables to analyze the difference between patients with and without low muscle mass in T2DM. Logistic regression analysis was used to evaluate the relationship between ALT/AST and low muscle mass. Multiple regression analysis was used to explore the correlation between ALT/AST, SMI, and other metabolic variables. *P* < 0.05 was considered to be statistically significant.

## 3. Results

### 3.1. Population Characteristics

The characteristics of subjects were shown in Table 1. The current study included 1068 adult patients with T2DM. Among all patients, 115 male patients (20.3%) and 71 female patients (14.1%) presented low muscle mass.

Subjects with low muscle mass had higher age, duration of diabetes, ALT/AST, HbA1c, and HDL, but lower BMI, DBP, ALT, AST, and TG compared with those with normal muscle mass in men. Low muscle mass group presented increased age, ALT/AST, and decreased BMI, FPG, and TG compared with normal muscle mass group in women.

### 3.2. The Correlation between ALT/AST and Low Muscle Mass

As shown in Table 2, ALT/AST showed an increased risk for low muscle mass in male (OR = 3.804) and female (OR = 3.320) patients with T2DM. After adjusting for age, smoking, drinking, and duration of diabetes, ALT/AST was still positively related to low muscle mass (OR = 2.763 for male and OR = 3.116 for female). Moreover, after adjusting age, duration of diabetes, drinking, smoking, SBP, DBP, FPG, HbA1c, TC, TG, HDL, and LDL, ALT/AST was still positively correlated with low muscle mass (OR = 2.179 for men and OR = 2.594 for women).

### 3.3. The Correlation between SMI and Clinical Variables

The correlation between SMI and clinical variables was shown in Table 3. SMI was negatively associated with age, ALT/AST, HbA1c, and HDL in men with T2DM. While in women with T2DM, SMI was positively related to SBP, and negatively related to age and ALT/AST.

### 3.4. The Correlation between ALT/AST and Other Characteristics

Table 4 showed the correlation of ALT/AST and other clinical variables. In male subjects, ALT/AST was positively related to age, but negatively related to BMI and TG. While in female subjects, ALT/AST was positively related to age whereas negatively related to BMI.

## 4. Discussion

Our research is aimed at investigating the relationship between ALT/AST and muscle mass in subjects with T2DM. The present results showed that ALT/AST was associated with an increased risk for low muscle mass in T2DM. ALT/AST was negatively correlated with SMI. In addition, ALT/AST was positively correlated with age and negatively correlated with BMI.

Age, physical inactivity, malnutrition, insulin resistance, and chronic inflammations are considered as main underlying mechanisms of low muscle mass or sarcopenia [17]. The present study showed that the age of the low muscle mass group was increased compared with the normal muscle mass group, which was in line with our expectation. Meanwhile, compared with subjects with normal muscle mass, the low muscle mass group showed lower BMI and TG in both genders. In the traditional view, obesity was regarded as a risk factor for low muscle mass or sarcopenia. Du et al. reported that several metabolic variables including BMI were associated with sarcopenia and skeletal muscle reduction [18]. Kadoma Sarcopenia Study performed in Japanese community-dwelling older adults indicated that obesity, hypertension, and malnutrition were risk factors for sarcopenia [19]. However, some other studies suggested different results. Senior et al. indicated that low BMI was a significant predictor of sarcopenia in populations living in retirement homes in Australia [20]. While in patients with T2DM, majority of previous researches implied that BMI was inversely related to sarcopenia. Chen et al. reported that sarcopenia was decreased with BMI and education, while increased with age in elderly individuals with T2DM in China [21]. Several relevant researches showed that patients with sarcopenia have lower BMI than those without sarcopenia in T2DM [22, 23]. In this study, our results implied that higher BMI was related to a declined risk for low muscle mass in T2DM. Previous literature showed that in T2DM, the TG level of subjects with sarcopenia was obviously lower than subjects without sarcopenia [24, 25]. We got similar results in our study. The precise mechanism underlying this association is unclear. Decreased TG levels perhaps implied a status of poor health accompanied with low muscle mass or sarcopenia [26].

ALT and AST are important serum biomarkers of human body which could be detected quickly, cheaply, and repeatedly worldwide. ALT mainly exists in liver, while AST can be expressed in heart, liver, skeletal muscle, kidney, erythrocyte, and brain. One study from Italy indicated that lower levels of ALT and AST were related to sarcopenia [27]. Another study suggested that low ALT activity was a useful predictor of sarcopenia and low whole body muscle mass [28]. Similar results were showed in our study. Our data indicated that ALT and AST was lower in patients with low muscle mass than in patients with normal muscle mass in male subjects. While in female subjects, ALT also presented a decreased tend in low muscle mass group compared with normal muscle mass group.

AST/ALT, also known as De Ritis ratio, was put forward firstly in 1957 by Fernando De Ritis, and used to estimate the severity of virus hepatitis [29]. After that, AST/ALT or ALT/AST has been applied not only in hepatic disorders but also gradually used in the assessment of cardiovascular diseases, chronic kidney diseases, and cancer [30–32]. In recent years, several studies indicated that liver enzymes had a positive association with insulin resistance. ALT/AST was considered as a good biomarker of insulin resistance in Asian populations [11, 33]. The association between ALT/AST and sarcopenia has rarely been reported before. He et al. reported that higher serum AST/ALT level was a risk factor for sarcopenia in populations aged over 50 years old in west China [15]. Our research showed different results. In our survey, higher level of ALT/AST was related to the reduction of muscle mass in T2DM patients. Considering that our research was conducted in patients with T2DM, who generally had decreased insulin secretion and insulin resistance, the contradictory results can be understood. Insulin can stimulate the synthesis of protein and inhibit its degradation, so insulin resistance and/or insufficient secretion may bring about insufficient protein, which will lead to low muscle mass or sarcopenia [34]. As ALT/AST is a good marker for insulin resistance, it can indicate the risk of low muscle mass or sarcopenia in patients with T2DM to some extent. Meanwhile, our study implied that ALT/AST was positively related to age and negatively related to BMI. The reason may be explained that the mass and metabolic activity of skeletal muscle decreased and the expression of AST relatively declined with the increase of age and the decrease of BMI.

The present study had several limitations. First of all, the sample size of this study was relatively small. Secondly, this study was a cross-sectional study, which limited its causal conclusions. Causality must be assessed by further longitudinal researches.

In short, ALT/AST is negatively associated with muscle mass among patients with T2DM.

## Figures and Tables

**Table 1:** The characteristics of patients with and without low muscle mass.

Characteristics	Male (*n* = 566)			Female (*n* = 502)		
	Normal muscle mass (*n* = 451)	Low muscle mass (*n* = 115)	*P* value	Normal muscle mass (*n* = 431)	Low muscle mass (*n* = 71)	*P* value
Age (years)	54.78 ± 11.95	60.69 ± 13.37	<0.001	61.11 ± 10.79	65.45 ± 11.36	0.002
Smoking			0.640			0.999
No (*n*)	211	51		426	70	
Yes (*n*)	240	64		5	1	
Drinking			0.932			0.999
No(*n*)	253	64		429	71	
Yes(*n*)	198	51		2	0	
Duration (years)	7.0 (4.0,10.0)	8.0 (6.0,12.0)	0.006	8.0 (5.0,10.0)	8.0 (5.0,10.0)	0.932
BMI (kg/m^2^)	27.53 ± 4.05	23.68 ± 4.89	<0.001	27.39 ± 4.59	22.33 ± 2.90	<0.001
SMI(kg/m^2^)	8.16 ± 1.50	6.45 ± 0.48	<0.001	6.50 ± 0.86	5.06 ± 0.33	<0.001
SBP (mmHg)	140.60 ± 18.54	137.31 ± 21.63	0.137	143.52 ± 20.83	142.76 ± 21.95	0.779
DBP (mmHg)	82.15 ± 12.47	78.54 ± 13.13	0.006	76.59 ± 12.63	76.58 ± 11.22	0.994
TG (mmol/L)	1.63 (1.05,2.46)	1.10 (0.85,1.83)	<0.001	1.47 (1.03,2.15)	1.32 (0.86,1.62)	0.002
TC (mmol/L)	4.49 ± 1.13	4.45 ± 1.08	0.724	4.70 ± 1.35	4.61 ± 1.16	0.624
LDL (mmol/L)	2.96 ± 0.89	2.95 ± 0.95	0.958	2.99 ± 0.93	2.90 ± 0.99	0.445
HDL (mmol/L)	1.14 ± 0.27	1.23 ± 0.34	0.011	1.29 ± 0.37	1.36 ± 0.35	0.136
FPG (mmol/L)	8.11 ± 2.78	7.96 ± 3.16	0.604	7.76 ± 2.89	6.99 ± 2.74	0.037
HbA1c (%)	8.39 ± 2.09	8.90 ± 2.16	0.028	8.33 ± 2.02	8.16 ± 2.15	0.528
ALT(U/L)	19.0 (14.0,28.0)	15.0 (11.0,21.0)	<0.001	16.0 (12.0,22.0)	14.0 (11.0,20.0)	0.066
AST(U/L)	17.0 (14.0,22.0)	16.0 (13.0,20.0)	0.003	16.0 (13.0,20.0)	18.0 (14.0,20.0)	0.402
ALT/AST	0.87 (0.68,1.07)	1.00 (0.82,1.26)	<0.001	1.00 (0.80,1.20)	1.09 (0.96,1.38)	<0.001

**Table 2:** The correlation between ALT/AST and low muscle mass.

Model	Male			Female		
	Model 1	Model 2	Model 3	Model 1	Model 2	Model 3
OR (95% CI)	3.804 (2.105-6.875)	2.763 (1.455-5.250)	2.179 (1.077-4.407)	3.320 (1.648-6.687)	3.116 (1.510-6.430)	2.594 (1.141-5.899)
*P* value	<0.001	0.002	0.030	0.001	0.002	0.023

Model 1 was crude model; model 2 was adjusted for age, duration of diabetes, drinking, smoking; model 3 was adjusted for age, duration of diabetes, drinking, smoking, SBP, DBP, FPG, HbA1c, TC, TG, HDL, and LDL.

**Table 3:** The correlation between SMI and clinical characteristics.

	Male		Female	
	*β* (95% CI)	*P* value	*β* (95% CI)	*P* value
Age (years)	-0.021 (-0.029, -0.013)	<0.001	-0.024 (-0.033, -0.015)	<0.001
Duration (years)	-0.002 (-0.017, 0.013)	0.784	0.005 (-0.009, 0.019)	0.475
SBP (mmHg)	0.004 (-0.001, 0.010)	0.094	0.006 (0.000, 0.011)	0.031
DBP (mmHg)	0.005 (-0.003, 0.013)	0.197	-0.001 (-0.010, 0.007)	0.754
ALT/AST	-0.270(-0.534,-0.006)	0.045	-0.396(-0.647,-0.146)	0.002
FPG (mmol/L)	0.024 (-0.010, 0.058)	0.164	-0.010 (-0.046, 0.026)	0.597
HbA1c (%)	-0.067 (-0.111, -0.023)	0.003	-0.016 (-0.065, 0.033)	0.520
TG (mmol/L)	0.016(-0.048, 0.080)	0.621	0.009 (-0.071, 0.088)	0.833
TC (mmol/L)	0.035(-0.255, 0.324)	0.814	0.028 (-0.175, 0.231)	0.787
LDL (mmol/L)	-0.015 (-0.307, 0.278)	0.921	-0.027 (-0.246, 0.192)	0.807
HDL (mmol/L)	-0.533 (-0.982, -0.084)	0.020	-0.266(-0.662, 0.131)	0.189

**Table 4:** The correlation between ALT/AST and clinical characteristics.

	Male		Female	
	*β* (95% CI)	*P* value	*β* (95% CI)	*P* value
Age (years)	0.008 (0.005, 0.010)	<0.001	0.003 (0.000, 0.007)	0.031
Duration (years)	0.002 (-0.003, 0.007)	0.469	0.002 (-0.003, 0.007)	0.483
BMI (kg/m^2^)	-0.007(-0.014,-0.001)	0.028	-0.010 (-0.016, -0.003)	0.002
SBP (mmHg)	0.000 (-0.001, 0.002)	0.675	-0.001(-0.003, 0.001)	0.292
DBP (mmHg)	0.000 (-0.003, 0.002)	0.876	-0.001 (-0.004, 0.002)	0.487
FPG (mmol/L)	-0.005 (-0.016, 0.006)	0.382	-0.002(-0.015, 0.011)	0.725
HbA1c (%)	0.004 (-0.010, 0.019)	0.566	-0.008(-0.025, 0.010)	0.403
TG (mmol/L)	-0.027(-0.048, -0.006)	0.013	-0.002(-0.030, 0.027)	0.916
TC (mmol/L)	0.045 (-0.049, 0.140)	0.348	-0.072(-0.145, 0.002)	0.055
LDL (mmol/L)	-0.044 (-0.139, 0.052)	0.372	0.040(-0.039, 0.119)	0.322
HDL (mmol/L)	0.088 (-0.059, 0.235)	0.243	0.132(-0.011, 0.275)	0.070

## Data Availability

Data are available upon reasonable request.
